# XIAP Knockdown in Alcohol-Associated Liver Disease Models Exhibits Divergent *in vitro* and *in vivo* Phenotypes Owing to a Potential Zonal Inhibitory Role of SMAC

**DOI:** 10.3389/fphys.2021.664222

**Published:** 2021-05-07

**Authors:** Li He, Tejasav S. Sehrawat, Vikas K. Verma, Amaia Navarro-Corcuera, Guneet Sidhu, Amy Mauer, Xin Luo, Tomohiro Katsumi, Jingbiao Chen, Soni Shah, Juan Pablo Arab, Sheng Cao, Hamid Kashkar, Gregory J. Gores, Harmeet Malhi, Vijay H. Shah

**Affiliations:** ^1^Division of Gastroenterology and Hepatology, Department of Internal Medicine, Mayo Clinic, Rochester, MN, United States; ^2^Department of Gastroenterology, Tongji Hospital, Tongji Medical College, Huazhong University of Science and Technology, Wuhan, China; ^3^Hepatic Surgery Center, Tongji Hospital, Tongji Medical College, Huazhong University of Science and Technology, Wuhan, China; ^4^Departamento de Gastroenterologia, Escuela de Medicina, Pontificia Universidad Catolica de Chile, Santiago, Chile; ^5^Centre for Molecular Medicine Cologne and Cologne Excellence Cluster on Cellular Stress Responses in Ageing-Associated Diseases, Institute for Medical Microbiology, Immunology and Hygiene, University of Cologne, Cologne, Germany

**Keywords:** ALD, apoptosis, scRNA sequencing, IAP, alcoholic hepatitis, alcohol-associated liver disease

## Abstract

Alcohol-associated liver disease (ALD) has been recognized as the most common cause of advanced liver disease worldwide, though mechanisms of pathogenesis remain incompletely understood. The X-linked inhibitor of apoptosis (XIAP) protein was originally described as an anti-apoptotic protein that directly binds and inhibits caspases-3, 7, and 9. Here, we investigated the function of XIAP in hepatocytes *in vitro* using gain and loss-of-function approaches. We noted an XIAP-dependent increase in caspase activation as well as increased inflammatory markers and pro-inflammatory EV release from hepatocytes *in vitro*. Primary hepatocytes (PMH) from *Xiap*^*Alb.Cre*^ and *Xiap*^*loxP*^ mice exhibited higher cell death but surprisingly, lower expression of inflammation markers. Conditioned media from these isolated *Xiap* deleted PMH further decrease inflammation in bone marrow-derived macrophages. Also, interestingly, when administered an ethanol plus Fas-agonist-Jo2 model and an ethanol plus CCl4 model, these animals failed to develop an exacerbated disease phenotype *in vivo*. Of note, neither *Xiap*^*Alb*^.^*Cre*^ nor *Xiap*^*AAV8.Cre*^ mice presented with aggravated liver injury, hepatocyte apoptosis, liver steatosis, or fibrosis. Since therapeutics targeting XIAP are currently in clinical trials and caspase-induced death is very important for development of ALD, we sought to explore the potential basis of this unexpected lack of effect. We utilized scRNA-seq and spatially reconstructed hepatocyte transcriptome data from human liver tissue and observed that *XIAP* was significantly zonated, along with its endogenous inhibitor second mitochondria-derived activator of caspases (SMAC) in periportal region. This contrasted with pericentral zonation of other IAPs including cIAP1 and Apollon as well as caspases 3, 7, and 9. Thus providing a potential explanation for compensation of the effect of *Xiap* deletion by other IAPs. In conclusion, our findings implicate a potential zonallydependent role for SMAC that prevented development of a phenotype in XIAP knockout mice in ALD models. Targeting SMAC may also be important in addition to current efforts of targeting XIAP in treatment of ALD.

## Introduction

Alcohol-associated liver disease (ALD) is one of the leading causes of chronic liver disease. Globally, approximately 2 million people die from chronic liver disease each year, of which 50% of deaths are attributable to alcohol use (Singal et al., 2018; Thursz et al., 2019; Sehrawat et al., 2020b). ALD comprises a spectrum of conditions arising due to excessive alcohol intake, ranging from isolated steatosis to acute alcohol-associated hepatitis, chronic fibrosis and cirrhosis and hepatocellular carcinoma (HCC) (Singal et al., 2018; Sehrawat et al., 2020b). Hepatocyte apoptosis has been recognized as a key mechanism of alcohol-induced liver injury (Feldstein and Gores, 2005; Gao and Bataller, 2011), with both the extrinsic, involving initiator caspase-8 (Khoruts et al., 1991; Galle et al., 1995; Taieb et al., 1998), and intrinsic, involving caspase-9, pathways (Kurose et al., 1997; Adachi et al., 2004) playing a role in ethanol-induced hepatocyte apoptosis. These pathways lead to the activation of the downstream effector caspases 3 and 7, which ultimately execute apoptosis (Lalaoui and Vaux, 2018).

X-linked inhibitor of apoptosis (XIAP) has been widely recognized as a potent anti-apoptotic protein. It acts by blocking the enzymatic activity of caspases 3, 7, and 9 via direct binding (Duckett et al., 1996; Liston et al., 1996; Uren et al., 1996), and is considered as the most potent caspase-binding protein and inhibitor of both the extrinsic and intrinsic apoptotic pathways (Holcik and Korneluk, 2001). Additionally, XIAP is also involved in inflammatory signaling pathways and cellular immune responses (Galban and Duckett, 2010; Jost and Vucic, 2019), development of Wnt signaling pathways (Hanson et al., 2012), and autophagy (Gradzka et al., 2018), relying on its capacity to promote protein ubiquitylation (Galban and Duckett, 2010). XIAP controls caspase activation in the steady state. In the event of excessive caspase activity-induced liver injury, such as ALD, endogenous IAPs fail to control caspase activation which leads to excessive cellular apoptosis (Feldstein et al., 2003). Therefore modulation of XIAP has been a compelling target in a variety of diseases, though not explored in ALD (Cao et al., 2004; Worthey et al., 2011; Zeissig et al., 2015; Hussain et al., 2017; Evans et al., 2018). Despite the known caspase-inhibitory function of XIAP, therapies harnessing the anti-caspase effects of XIAP are lacking due to poor understanding of its regulation and the ability to selectively modulate XIAP in hepatocytes. In this study, we identified a potential protective role of XIAP against alcohol-induced hepatocyte injury using *in vitro* models. We then sought to evaluate the effect of hepatocyte-specific XIAP deletion in two mice models of ALD. Interestingly, we observed a lack of effect of XIAP deletion in these models. We demonstrated a potential role of second mitochondria-derived activator of caspases (SMAC), an endogenous inhibitor of XIAP, in this divergence utilizing single cell RNA sequencing (scRNA-seq) data.

## Materials and Methods

### Animal Studies

All animal experiments were approved by the Institutional Animal Care and Use Committee (IACUC) and were performed in accordance with the National Institutes of Health guidelines. All mice were bred and maintained in the animal center at Mayo Clinic, Rochester.

### Cell Culture

HCC cell line HepG2^*Cyp2E1*^ were routinely cultured in 10% FBS supplemented DMEM. The HepG2^*Cyp2E1*^ cells were a kind gift from Dr. D. Clemens (University of Nebraska Medical Center, United States) and have been described earlier by us and others and overexpress alcohol metabolizing enzyme cytochrome P450 2E1 (Donohue et al., 2006; Liu et al., 2017). Cells were cultured in Dulbecco’s Modified Eagles Media (DMEM, Life Technologies) supplemented with 10% fetal bovine serum (FBS), glucose (4.5 g/L), penicillin (100 units/mL), and streptomycin (100 μg/mL). Cells were cultured in a reduced extracellular vesicle (EV) medium and stimulated with ethanol (50 mM or 100 mM) as indicated in individual experiments. For *in vitro* studies, BV-6 (4 μM) was added with ethanol for 24 h.

### Primary Mouse Hepatocyte Isolation

Murine primary hepatocytes were isolated as described earlier (Malhi et al., 2006; Dasgupta et al., 2020). Briefly, a 2-step collagenase perfusion technique was performed. After in situ perfusion of the liver with a calcium-free HBSS medium (Corning, Corning, NY, United States) followed by a collagenase D (Roche, Indianapolis, IN, United States) solution in HBSS medium with calcium and magnesium (Corning, Corning, NY, United States), livers were excised and minced in Krebs-Henseleit buffer (Sigma-Aldrich, St. Louis, MO, United States). Dispersed cell suspensions were purified by Percoll solution (Sigma). The hepatocytes were cultured in Dulbecco’s modified Eagle’s medium (DMEM, Life Technologies) containing 10% fetal bovine serum and 1% penicillin/streptomycin (Gibco) in collagen coated plates, prepared with Collagen coating solution (Sigma-Aldrich, St. Louis, MO, United States). Approximately, 2.5×10^6^ cells were plated in 10 cm dishes, 1.5×10^6^ cells were plated in 60 mm dishes, and 750,000 cells per well in 6-wells plates.

### Bone Marrow-Derived Macrophages Isolation

Bone marrow–derived macrophages (BMDMs) were isolated from the hind legs of C57BL/6J mice and used for *ex vivo* experimentation, as previously described (Liao et al., 2018). Once euthanized, the mouse was sprayed with 70% ethanol, and the skin was cut open using sterile scissors to expose the hind legs. Cuts were made through the hip and ankle joints to remove each leg. The legs were placed in 70% ethanol for 2–3 min, then placed in phosphate-buffered saline (PBS) on ice. In the tissue culture hood, the leg muscle and epiphyses were removed and bone marrow flushed out onto a petri dish using a syringe and 25 gauge needle containing BMDM media. The BMDM media used for bone marrow differentiation consists of Roswell Park Memorial Institute (RPMI)-1640 supplemented with 20% L929 cell-conditioned medium (LCM), 10% fetal bovine serum (FBS), penicillin (100 units/mL) and streptomycin (100 μg/mL). The flushed media containing bone marrow was drawn through a 23 gauge needle 4–5 times to remove clumps. Bone marrow cells were plated onto 150 mm petri dishes (BD Falcon, Oxford, United Kingdom) and incubated at 37°C, 5% CO_2_. BMDM media was changed every 2 days on Day 3 and Day 5, and BMDMs were dissociated with Accutase and used in experiments on Day 7.

### Isolation of Extracellular Vesicles

Reduced EV media was prepared by ultracentrifugation of 20% FBS containing DMEM for 2 h using a SW32Ti Rotor at 100,000 *g*, 4°C in Optima XPN-80 ultracentrifuge. Cells were cultured in an EV free 10% FBS medium. EVs were then isolated from cultured cells or human plasma samples as described earlier (Sehrawat et al., 2020a). Supernatants from cultured cells were subjected to differential ultracentrifugation for 45 mins (20,000 *g*) and twice for 2 hrs (110,000 *g*) at 4°C. Determination of EV concentration and size was completed using nanoparticle tracking analysis (NTA) (NS300, Malvern Instruments, Malvern, United Kingdom).

### Isolation of Human PBMCs and M1-Like Macrophage Derivation

Primary macrophage experiments were carried out using monocyte-derived macrophages (MDM), obtained from peripheral blood mononuclear cells (PBMC) isolated from whole blood using ficoll density gradients as described by us previously (Verma et al., 2016). PBMC were plated and monocytes were allowed to attach on the non-coated 12 well plates for 4 h. Attached cells were washed twice with PBS and cultured in complete RPMI medium with 50 ng/ml monocyte colony stimulating factor (M-CSF) for 5 days. Fresh M-CSF containing medium was replaced on Day 3. On Day 5 medium was replaced with complete medium and MDM were used for experiments on Day 6. The cells were treated with equal volume of EV derived from healthy and heavily drinking controls or alcoholic hepatitis patients for 12 h.

### Study Subjects and Blood Samples

The blood samples used in this study were collected from 3 healthy controls, heavily drinking controls and alcoholic hepatitis patients enrolled into the multicenter prospective Translational Research and Evolving Alcoholic Hepatitis Treatment Study (TREAT001, NCT02172898) at Mayo Clinic, United States. The study was also approved by the Institutional Review Board (IRB# 13-002715; PI: VS).

### Generation and Selection of XIAP Knockdown (KD), Knockout (KO), and Overexpression Cells

We utilized both short-hairpin (shRNA) based knock-down and CRISPR-CAS9 based complete knockout cells to exclude the clonal artifacts associated with the method employed. We packaged three targeted shRNA constructs with different sequences into the lentivirus-based system for efficient transfection and selection. These XIAP shRNA packaged lentiviruses were then transfected into HepG2^*Cyp2E1*^ cells, which were later selected with puromycin. CRISPR/cas9 mediated gene editing technology was used to generate knockout cells using Guide-it CRISPR-Cas9 technology. Three target specific guide RNA sequences; gRNA1, gRNA2, and gRNA3; were designed based on the sequence of exon 1 of XIAP. This was done utilizing the tools available at crispr.mit.edu. The CDS sequence was verified from published NCBI database. Following transfection of GuideRNA integrated vector, a single cell-derived clone of cells was obtained by limiting dilution method. DNA was extracted from a total of 15 monoclonal cell lines to perform PCR. Following PCR, clones containing the deletion associated frameshift mutation and the clones containing insertions in XIAP gene were screened and then confirmed to have disrupted XIAP gene function. The gain of function of XIAP was achieved by transfection of an expression plasmid vector containing full-length XIAP.

### Generation of Hepatocyte-Specific XIAP Deletion Mice

X-linked inhibitor of apoptosis floxed mice (XIAP^*loxP*^) and albumin Cre mice (Alb^*Cre*^) were crossed to generate offsprings with hepatocyte-selective deletion of XIAP (XIAP^*Alb.Cre*^) (Andree et al., 2014). Mice were genotyped using a polymerase chain reaction (PCR) based approach. Adeno-associated virus type 8 (AAV8)-cre or AAV8-vector (Penn Vector Core, Philadelphia, PA, United States) was injected (10^11^ viral genome copies/mouse, intravenously injected) in XIAP^*loxP*^ mice to drive hepatocyte-specific expression of cre. Mice were studied 4 weeks after AAV8-cre or AAV8-vector injections. The efficiency of XIAP deletion in hepatocytes was assessed using qPCR or Western blotting of mice liver tissue.

### Chronic Ethanol Feeding Plus Jo2 Mice Model

C57BL/6J XIAP floxed mice (XIAP f/f) and hepatocyte specific XIAP knockout (XIAP KO) mice (Alb^*Cre*^/XIAP^*loxP*^) weighed 19.8 ± 2.4 g at 6–8 weeks of age. The XIAP f/f (*n* = 17) and XIAP KO (*n* = 23) mice were pair-fed a control dextrose (Dex) diet or liquid ethanol (EtOH) diet (Bio-Serv, Frenchtown, NJ, United States) with 2% and 4% ethanol (vol/vol), each for 5 days, and at 5% for 28 days. After the 4-week feeding period, the body weight of the pair-fed XIAP f/f and XIAP KO mice was 20–24 g (gain of 2–3 g body weight), whereas the body weight of the ethanol-fed XIAP f/f and XIAP KO mice was 20.1 ± 2.7 g. The ethanol and food were then withdrawn, and the mice received one dose of 0.1 μg of Jo2 anti-Fas antibody (Jo2) (BD Pharmingen, San Diego, CA, United States) or saline control (Sal) intraperitoneally. Eight hours after administration of Jo2 or Sal, the mice were sacrificed and serum and liver samples were collected (Wang et al., 2009).

### Chronic Ethanol Feeding Plus CCl4 Mice Model

At 6–8 weeks of age, XIAP f/f mice were injected with AAV8-null (*n* = 14) or AAV8-CRE (*n* = 14). Four weeks later, mice were gradually started on a pair-fed control Dex diet or EtOH diet (Bio-Serv, Frenchtown, NJ, United States) with 2% and 4% ethanol (vol/vol), each for 5 days, and at 5% for 42 days. During this time 0.5 μl/kg carbon tetrachloride (CCl4) or corn oil was given every 3rd day for 6 weeks. Two days after administration of CCl4 or corn oil, the mice were sacrificed, and serum and liver samples were collected. During ethanol feeding and CCl4 injection, 6 mice died. The final ethanol plus CCl4 injection group included 3 AAV8-null mice and 5 AAV8-CRE mice.

### RNA Preparation and Quantitative PCR

An RNeasy kit (QIAGEN, Germantown, MD) was used to extract total RNA from the liver tissue according to the manufacturer’s instructions. 500 ng of mRNA was used for cDNA synthesis with dNTP and oligo primer using SuperScript III (Invitrogen, Waltham, MA, United States) first-strand synthesis system for reverse transcription PCR (RT-PCR) per the manufacturer’s protocol. Real-time PCR was performed with the same amount of cDNA in a total 25-μl-volume reaction using iQ SYBR Green supermix (Bio-Rad, Hercules, CA, United States) and the QuantStudio 3 Real-Time PCR System (Thermo Fisher Scientific, Waltham, MA, United States) in accordance with the manufacturer’s instructions. Amplification of β-actin was performed in the same reaction for respective samples as internal control. Each experiment was performed in triplicate. The cycling conditions were as follows: 2 min at 50°C and 10 min at 95°C followed by 39 cycles at 95°C for 15 s, 60°C for 1 min, 95°C for 10 s, and 65°C for 5 s. Primers used for this study are listed in Table 1. Levels of mRNA were calculated using the 2^–ΔΔ*Ct*^ threshold cycle method and normalized to those of β-actin mRNA.

**TABLE 1:** Mouse primers used in RT-qPCR analysis.

Gene	Forward Primer (5′-3′)	Reverse Primer (5′-3′)
*Xiap*	TCACAGCACTCCAACTCTAATC	GACCTTCCGAGTGACCATTT
*Smac*	CCTACCTGCGTGAAGATTGAG	GCAGAGCTGGGACAACATTA
*b-actin*	CCTCCCTGGAGAAGAGCTATG	TTACGGATGTCAACGTCACAC
*Tnfa*	CTACCTTGTTGCCTCCTCTTT	GAGCAGAGGTTCAGTGATGTAG
*Il-1b*	ATGGGCAACCACTTACCTATTT	GTTCTAGAGAGTGCTGCCTAATG

### Western Blot

Cells were lysed in RIPA buffer (Cell Signaling Technology, Danvers, MA, United States). The samples were mixed with 6 × loading buffer (375 mM Tris.HCl, 9% SDS, 50% Glycerol, 0.03% Bromophenol blue) and boiled for 10 min, and proteins were separated by SDS-PAGE as described earlier (Navarro-Corcuera et al., 2019). The separated proteins in the gels were electrophoretically transferred onto a nitrocellulose membrane at 100 V for 120 min. The blotted membrane was probed with anti-XIAP, anti-caspase 3 and anti-cleaved-caspase 3 (1:1000, 1:500 and 1:500, respectively, Cell Signaling Technology), anti-GAPDH (1:5000; Thermo Fisher Scientific), and anti-HSC70 (1:2000, Santa Cruz Biotechnology). Immunoreactive bands were visualized using horseradish peroxidase-conjugated secondary antibody and the enhanced chemiluminescence system (Santa Cruz Biotechnology). All experiments are representative of a minimum of three independent experiments with quantification and statistics performed using ImageJ and Prism 5, respectively.

### Caspase 3/7 Activity

The assay was performed as described by us previously (Malhi et al., 2006). Cells were plated in 96-well plates (Corning Inc., Corning, NY, United States). Caspase activity assay was performed using the commercially available Apo-ONE homogeneous caspase 3/7 assay (Promega Corp.) according to the manufacturer’s instructions. Briefly, this assay involves cleavage of a profluorescent caspase 3/7 consensus substrate, bis-(*N*-benzyloxycarbonyl-L-aspartyl-L-glutamyl-L-valyl-aspartic acid amide) conjugated to rhodamine 110 (Z-DEVD-R110) on its carboxyl-terminal side. Proteolytic cleavage liberates rhodamine 110, unquenching its fluorescence. Fluorescence was measured using excitation and emission wavelengths of 498 and 521 nm, respectively.

### Annexin V-FITC/Propidium Iodide Assay

Annexin V-propidium iodide (PI) staining was carried out with an APC FITC-Annexin V Apoptosis Detection Kit with PI (#640932; BioLegend, San Diego, CA, United States). Primary mouse hepatocytes were plated in a 96 well plate (Corning^®^ 3603, ME, United States) at a concentration of 5,000 cells per well. The cells were incubated in 100 μl staining media with FITC-Annexin V and PI for 45 mins at 37°C with 5% CO2 in the dark according to manufacturer’s instructions. The samples were analyzed on a Celigo S Image Cytometer (Nexcelom Bioscience, United States).

### Imaging Assays

Mice livers were sectioned using a Leica cryostat. TUNEL staining was performed on 5 μm cryosections after fixation with 4% paraformaldehyde followed by permeabilization by 0.1% Triton X-100 and 0.1% sodium citrate for 2 min on ice before staining. For TUNEL staining, the In Situ Cell Death Detection Kit, Fluorescein (Roche) was utilized according to the manufacturer’s instructions. The samples were visualized and quantified using previously described protocols (Cao et al., 2010). Oil-Red O staining was performed with 8 μm sections using a previously published protocol (Arab et al., 2020a). Sirius-red staining and immunohistochemistry were performed on mouse liver tissue sections after fixation in 10% neutral buffered formalin and embedding in paraffin according to previously described protocols (Cao et al., 2010).

### Single-Cell RNA Sequencing Analysis

The normalized gene expression matrices for single cells were downloaded and further analyzed from GEO^1^. The source data was obtained from GEO Accession No. GSE146409^2^. The original scRNA-seq experiment was performed as described by Massalha et al. (2020). Briefly, MARS-seq libraries were prepared as described previously (Jaitin et al., 2014). scRNA-seq analysis was performed using Seurat 3.1 (Satija et al., 2015). Annotation for clusters was done based on highly expressed genes for each cluster. Hepatocyte zonation analysis was performed using and as described in the algorithm described in Massalha et al. (2020).

### Alanine Aminotransferase (ALT) and Triglyceride Assay

Serum ALT levels were assayed using a diagnostic kit (ScienCell Research Laboratories). Hepatic triglyceride content was assessed according to methods described by us before (Arab et al., 2020a).

### Statistical Analysis

Results were expressed as means ± SE from three or more independent experiments. Two-tailed Student’s t-test or ANOVA was used to test for statistical significance between groups as appropriate. A P value of < 0.05 was considered as statistically significant.

## Results

### XIAP Is Protective Against Ethanol-Induced Caspase Activation and Inflammation *in vitro*

**FIGURE 1:**
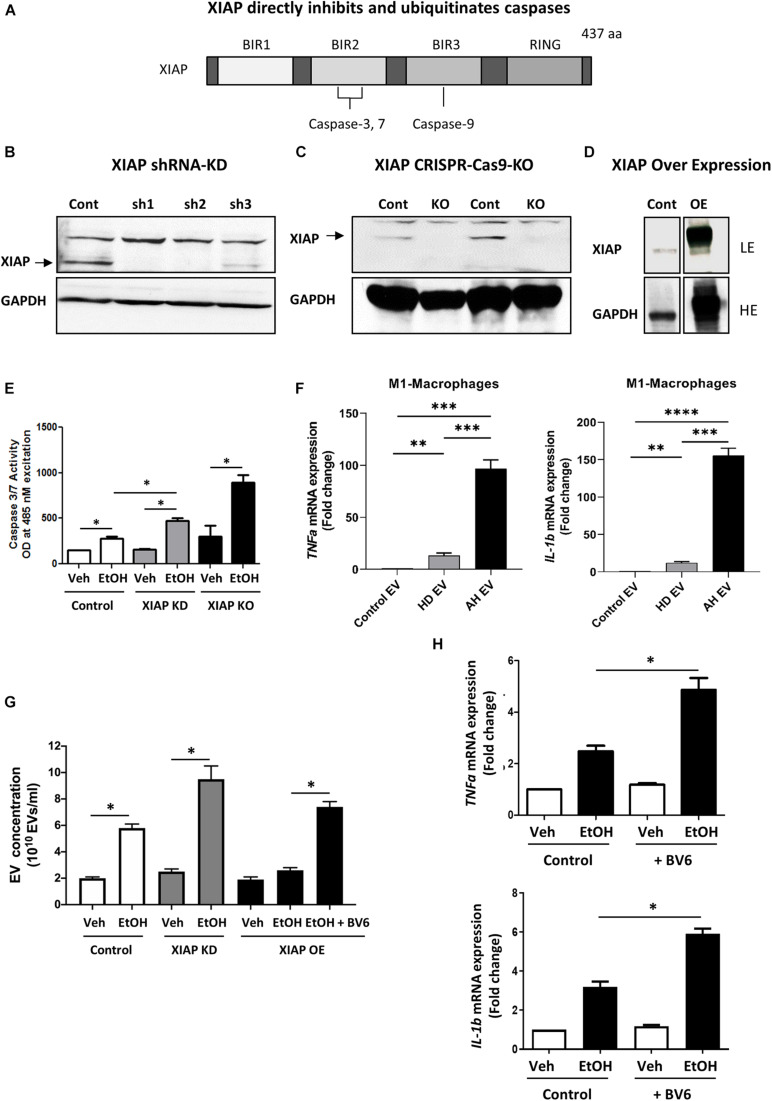
*XIAP* knockdown potentiates ethanol-induced apoptosis and inflammation in HepG2^*Cyp2e1*^ cells. **(A)** Scheme shows *XIAP* gene structure containing caspase-binding sites. **(B)** Protein levels of XIAP determined by Western blot in HepG2^*Cyp2e1*^ transfected with non-targeted shRNA (Cont) or shXIAP (sh). **(C)** Protein levels of XIAP determined by Western blot in control HepG2^*Cyp2e1*^ (Cont) and XIAP CRISPR-Cas9-KO HepG2^*Cyp2e1*^ (KO). **(D)** Protein levels of XIAP determined by Western blot in HepG2^*Cyp2e1*^ transfected with empty vector (Cont) or with XIAP plasmid (overexpression, OE) (LE, low exposure; HE, high exposure). **(B–D)** GAPDH protein levels were used as a loading control for total protein. **(E)** Evaluation of Caspase 3/7 activity in control HepG2^*Cyp2e1*^ (Control), HepG2^*Cyp2e1*^ transfected with shXIAP (XIAP KD), and in XIAP CRISPR-Cas9-KO HepG2^*Cyp2e1*^ (XIAP KO) treated with vehicle (Veh) or with 50 mM ethanol (EtOH). **(F)** Effect of EVs isolated from AH patients in mRNA expression of *TNFa* (left panel) and *IL-1b* (right panel) in M1 macrophages determined by qRT-PCR. **(G)** EV release counting in control HepG2^*Cyp2e1*^ (Control), HepG2^*Cyp2e1*^ transfected with shXIAP (XIAP KD), and in XIAP-vector-transfected HepG2^*Cyp2e1*^ (XIAP OE) treated with vehicle (Veh) or with 50 mM ethanol (EtOH). **(H)** Relative mRNA levels of *TNFa* (left panel) and *IL-1b* (right panel) determined by qRT-PCR and expressed as fold change. *GAPDH* was used as a housekeeping gene (^∗^*p* < 0.05; ^∗∗^*p* < 0.01; ^∗∗∗^*p* < 0.005; and ^****^p < 0.001, *n* = 3).

XIAP is a potent inhibitor of apoptosis that directly inhibits and ubiquitinates caspases, importantly BIR2, BIR3 and the linker domain between the two (Figure 1A). We utilized gain and loss of function approaches to understand the role of XIAP in ethanol treatment using *in vitro* models. Immunoblotting analysis for XIAP expression from the selected shRNA clones (XIAP-KD cells) was carried out demonstrating efficiency in HepG2^*Cyp2E1*^ cells compared with non-targeted shRNA used as control (Figure 1B). Furthermore, following sequencing confirmation of CRISPR-Cas9 clones (XIAP-KO cells), the complete loss of XIAP protein in cell lysates was verified with an immunoblotting analysis using 2 clones of cells (Figure 1C). To confirm functional overexpression following specific plasmid transfection, cell lysates from XIAP-OE cells were subjected to immunoblotting analysis. A significant increase in XIAP protein expression was noted when compared with both control and XIAP-KD cells (Figure 1D). These cells were then used for subsequent caspase activation assays and EV release experiments.

We treated HepG2^*Cyp2E1*^ cells with 50 mM concentration of ethanol for 24 hrs. Following treatment, the activation of caspase 3/7 was measured using a sensitive spectrofluorometric method. Interestingly, HepG2^*Cyp2E1*^ cells showed a significant increase in caspase 3/7 activity with 50 mM concentration of ethanol in XIAP-KD cells compared with controls (Figure 1E). Furthermore, this ethanol-induced caspase activation was much more pronounced in XIAP-KO cells than both XIAP-KD and control cells (Figure 1E).

In order to examine the potential role of XIAP on inflammation we used ethanol-induced EV release as a marker for inflammation (Verma et al., 2016; Arab et al., 2020b). We isolated PBMCs from AH subjects and differentiated them to M1-like macrophages. We then used EVs isolated from healthy and heavily drinking controls, as well as AH subjects (*n* = 3 each) to treat these M1-like macrophages. We validated the pro-inflammatory nature of these EVs using qPCR and observed a significant increase in TNF-α and IL-1β expression in macrophages treated with EVs from AH patients (Figure 1F). Once validated, we used HepG2^*Cyp2e1*^ cells to understand the effect of XIAP manipulation on this pro-inflammatory process. As expected, we noted a significant increase in EV release with ethanol treatment (Verma et al., 2016; Sehrawat et al., 2020a). Interestingly, this EV release was increased in XIAP-KD cells and conversly attenuated with XIAP overexpression (Figure 1G). When cells were pre-treated with a pharmacological inhibitor of XIAP (BV6), EV release was significantly higher (Figure 1G). In conjunction with these results, BV6 treatment also resulted in a significantly higher mRNA expression of TNF-α and IL-1β (Figure 1H).

Together these results point towards a potential role of XIAP in reducing alcohol-induced hepatocyte cell death and inflammation in our *in vitro* model using HepG2^*Cyp2E1*^ tumoral hepatocyte cell line. These data prompted us to investigate the role of this important IAP using an *ex vivo* model of ethanol-induced hepatocyte injury.

### XIAP Deficiency Exacerbates Cell Death but Protects Against Inflammation in Primary Mouse Hepatocytes

**FIGURE 2:**
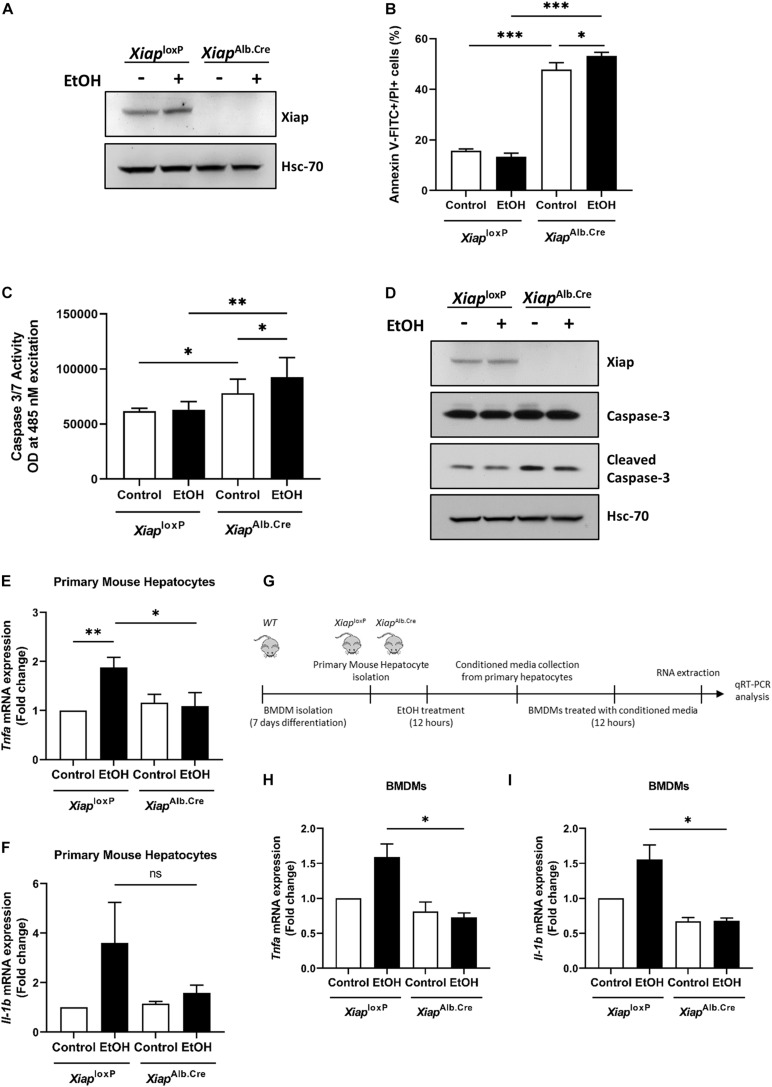
Hepatocyte-selective knockout of *Xiap* modulates apoptosis and inflammation in PMH. **(A)** Evaluation of *Xiap* deletion in primary hepatocytes isolated from *Xiap*^*loxP*^ and *Xiap*^*Alb.Cre*^ mice after 12 h EtOH treatment. Xiap protein levels were determined by Western blot. Hsc-70 protein expression was used as a loading control for total protein. **(B–D)** Evaluation of the effect of *Xiap* deletion in apoptosis by analyzing Anexin V-FITC+PI+ **(B)**, Caspase 3/7 activity **(C)**, and cleaved Caspase-3 protein expression **(D)** in primary hepatocytes isolated from *Xiap*^*loxP*^ and *Xiap*^*Alb.Cre*^ mice following EtOH treatment for 12 h. **(E,F)** Analysis of the effect of *Xiap* deletion in inflammation by quantifying relative mRNA levels of *Tnfa* **(E)** and *Il-1b* **(F)** by qRT-PCR in primary hepatocytes isolated from *Xiap*^*loxP*^ and *Xiap*^*Alb.Cre*^ mice following EtOH treatment for 12 h. *Gapdh* was used as a housekeeping gene. **(G)** Schematic work flow for *PMH* experiments showed in panel **(H)** and panel **(I)**. **(H,I)** qPCR analyses of the relative mRNA levels of *Tnfa* **(H)** and *Il-1b* **(I)** in BMDMs incubated with conditioned media collected from *Xiap*^*loxP*^- and *Xiap*^*Alb.Cre*^ mice-primary hepatocytes treated with or without EtOH. *Gapdh* was used as a housekeeping gene (^∗^*p* < 0.05; ^∗∗^*p* < 0.01; ^∗∗∗^*p* < 0.005; ns, no significance, and *n* = 3).

As HepG2^*Cyp2E1*^ is a tumoral cell line, we wanted to understand the role of XIAP in primary mouse hepatocytes (PMH) treated with ethanol on cell death and inflammation. Therefore, we isolated PMH from *Xiap*^*loxP*^ and *Xiap*^*Alb.Cre*^ animals and bone marrow derived macrophages (BMDMs) from wild-type mice with the same genetic background. We first verified the efficacy of Xiap deletion in PMH from *Xiap*^*Alb.Cre*^ animals. Xiap protein levels were completely absent in lysates from these PMH (Figure 2A).

To evaluate effect of XIAP modulation on cell death, we first treated PMH from *Xiap*^*loxP*^ and *Xiap*^*Alb.Cre*^ with vehicle or ethanol (100 mM) for 16 h and then dual labeled with Annexin V-FITC and PI and observed a significant increase in early and late apoptosis in PMH from *Xiap* deleted mice basally, and this was further significantly exacerbated when these cells were treated with ethanol (Figure 2B). We also performed a spectrophotometric assay of caspase 3/7 activity and observed a similar increase in caspase activation in *Xiap*^*Alb.Cre*^ mice basally and a further exacerbation on treatment with ethanol (Figure 2C). Western blotting for cleaved caspase-3 validated these findings at the protein level (Figure 2D). To evaluate the effect of *Xiap* deletion on inflammation, we first treated PMH from *Xiap*^*loxP*^ and *Xiap*^*Alb.Cre*^ mice with vehicle or ethanol (100 mM) for 12 h. We noted an expected increase in *Tnf-α* and *Il-1β* expression levels in cells treated with ethanol. Interestingly, the expression levels were decreased in PMH from *Xiap* deleted mice (Figures 2E,F). We then used conditioned media from these cells to treat BMDMs as shown in the scheme (Figure 2G). Interestingly, *Tnf-α* and *Il-1β* expression levels were reduced in BMDMs treated with conditioned media from *Xiap*^*Alb.Cre*^ PMH treated with or without ethanol (Figures 2H,I).

These results showed a divergent impact of *Xiap* deletion on disease process in PMH when exposed to alcohol-induced injury. As *Xiap* deletion exacerbated apoptotic cell death but seemed to protect against inflammation we decided to assess role of this deletion *in vivo*.

### Hepatocyte-Specific Genetic Knockout of XIAP Does Not Exacerbate Injury in EtOH Plus Jo2 Mouse Model

**FIGURE 3:**
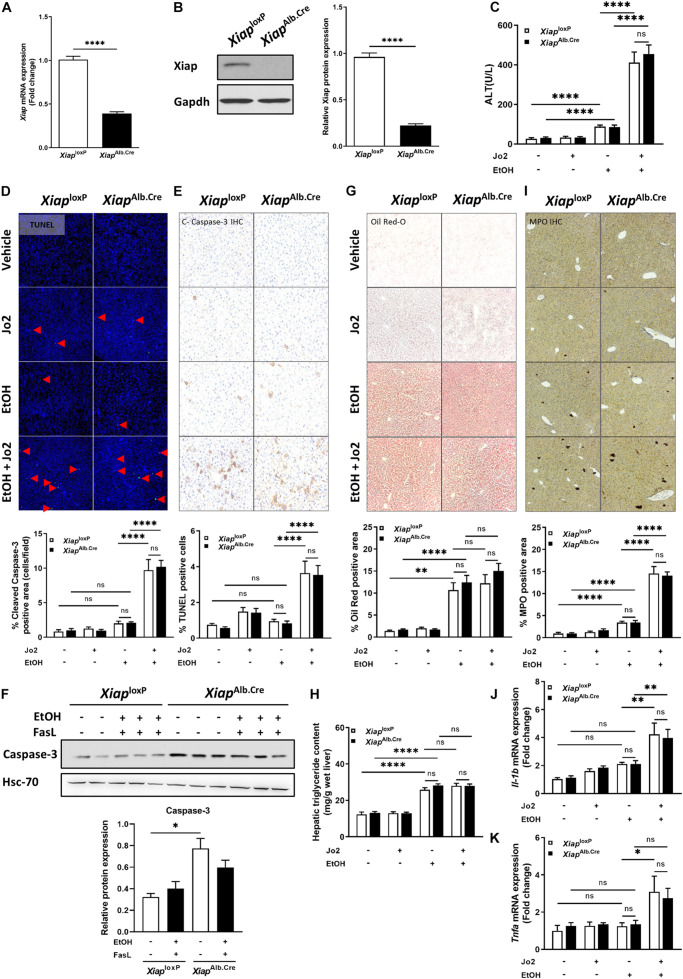
Hepatocyte-specific genetic knockout of *Xiap* does not exacerbate injury in EtOH plus Jo2 mouse model. **(A)** Relative mRNA levels of *Xiap* determined by qRT-PCR in *Xiap*^*loxP*^- and *Xiap*^*Alb.Cre*^ mice and expressed as fold change. *β-actin* was used as a housekeeping gene. **(B)** Protein levels of XIAP determined by Western blot in *Xiap*^*loxP*^- and *Xiap*^*Alb.Cre*^ mice. GAPDH expression was used as a loading control for total protein. **(C)** Serum ALT levels from pair-fed and ethanol-fed mice with and without Jo2 administration. **(D,E,G,I)** Histological evaluation of the role of Xiap in liver tissues collected from *Xiap*^*loxP*^- and *Xiap*^*Alb.Cre*^ mice that underwent ethanol or paired-feeding and were administered saline or Jo2. TUNEL **(D)**, cleaved-Caspase-3 IHC **(E)**, Oil Red-O staining **(G)**, and MPO IHC **(I)** were performed. Representative images and quantifications from mice are shown. Image magnification 10X. **(F)** Protein expression of Caspase-3 determined by Western blot in liver tissue from *Xiap*^*loxP*^- and *Xiap*^*Alb.Cre*^ mice that underwent ethanol or paired-feeding. Representative image is shown. Hsc-70 protein levels were used as a loading control for total protein. **(H)** Hepatic triglyceride (TG) levels expressed as mg/g of wet liver weight. **(J,K)** Relative mRNA levels of *Il-1b* **(J)** and *Tnfa* **(K)** *as* determined by qRT-PCR in *Xiap*^*loxP*^- and *Xiap*^*Alb.Cre*^ mice and expressed as fold change. *b-actin* was used as a housekeeping gene (^∗^*p* < 0.05; ^∗∗^*p* < 0.01; ^∗∗∗^*p* < 0.005; ^****^p < 0.001; ns, no significance, and *n* = 3–6).

To elucidate the role of XIAP in ALD, we utilized a hepatocyte-specific (Albumin-Cre) genetic deletion model of XIAP. *Xiap* deletion was confirmed in liver tissue using qPCR (Figure 3A) and Western blotting (Figure 3B). In the chronic ethanol feeding plus Jo2 mice model, serum ALT was elevated approximately 5 times after EtOH and Jo2 administration (*p* < 0.001) (Figure 3C). However, there was no significant difference between *Xiap*^*loxP*^ and *Xiap*^*Alb.Cre*^ mice in the EtOH+Jo2 group (*p* = 0.55). Chronic ethanol feeding plus Jo2 also increased programmed cell death. TUNEL and cleaved caspase 3 staining showed increased apoptotic hepatocytes in the ethanol and Jo2 administered mice as compared with controls (*p* < 0.01), but there was no significant difference between *Xiap*^*loxP*^ and *Xiap*^*Alb.Cre*^ EtOH+Jo2 groups (Figures 3D,E). We observed the same effects with Western blotting for caspase-3 levels (Figure 3F). Oil Red-O staining showed that mice fed with the ethanol diet developed significant steatosis compared to pair-fed mice (*p* < 0.05) (Figure 3G), but there was no difference between the *Xiap*^*loxP*^ and *Xiap*^*Alb.Cre*^ EtOH fed groups (*p* = 0.31). This was mirrored by hepatic triglyceride levels (Figure 3H). MPO staining increased after treatment of EtOH+Jo2 in both *Xiap*^*loxP*^ and *Xiap*^*Alb.Cre*^ mice (*p* < 0.05) (Figure 3I). However, there was no significant difference between *Xiap*^*loxP*^ and *Xiap*^*Alb.Cre*^ EtOH+Jo2 groups (*p* = 0.77). The same was noted by qPCR for pro-inflammatory cytokines TNF-α and IL-1β between *Xiap*^*loxP*^ and *Xiap*^*Alb.Cre*^ mice (Figures 3J,K). As such, these results indicate that hepatocyte XIAP deletion does not aggravate liver injury, steatosis or inflammation in EtOH plus Jo2 mice model.

### Hepatocyte-Specific AAV8-TBG.CRE Mediated Deletion of XIAP Does Not Exacerbate Injury in EtOH Plus CCl4 Mouse Model

**FIGURE 4:**
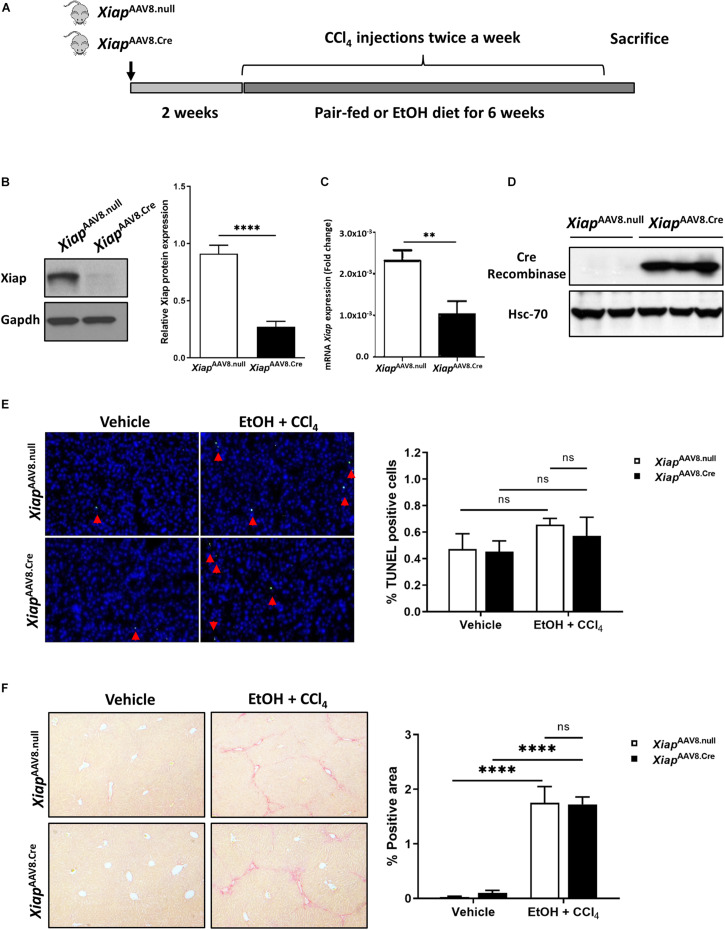
Hepatocyte-specific AAV8 deletion of *Xiap* does not exacerbate injury in EtOH plus CCl4 mouse model. **(A)** Schematic representation of the mouse model. **(B)** Protein levels of Xiap determined by Western blot in *Xiap*^*AAV8.null*^ and *Xiap*^*AAV8.Cre*^ mice. Gapdh protein levels were used as a loading control for total protein. **(C)** Evaluation of *Xiap* deletion by qRT-PCR in *Xiap*^*AAV8.null*^ and *Xiap*^*AAV8.Cre*^ mice. *Xiap* mRNA levels were expressed as fold change. *Gapdh* was used as a housekeeping gene. **(D)** Protein levels of Cre-Recombinase determined by Western blot in *Xiap*^*AAV8.null*^ and *Xiap*^*AAV8.Cre*^ mice. Hsc-70 protein expression was used as a loading control for total protein. **(E,F)** Histological evaluation of the role of *Xiap* in liver tissues collected from *Xiap*^*AAV8.null*^ and *Xiap*^*AAV8.Cre*^ mice that underwent ethanol or paired-feeding and were administered olive oil or CCl_4_. TUNEL **(E)** and Sirius red staining **(F)** were performed. Representative images and quantifications from mice liver tissues are shown. Image magnification 10X (^∗∗^*p* < 0.01; ^****^p < 0.001; ns, no significance, and *n* = 3–7).

Next, we used the EtOH plus CCl4 mice model, which showed greater necrosis and fibrosis compared to EtOH administration in absence of CCL4 (Furuya et al., 2016). In this model, we generated hepatocyte-specific *Xiap* knockout mice by AAV8-TBG.Cre virus intravenous injection to *Xiap*^*loxP*^ mice (Figure 4A). The efficiency of *Xiap* deletion by AAV8.Cre virus was confirmed using Western blotting and qPCR for Xiap and Cre-Recombinase (Figures 4B–D). Chronic ethanol feeding plus CCl4 did not increase TUNEL staining compared with the control diet plus olive oil group (p > 0.3) (Figure 4E). There was no statistically significant difference in Sirius Red staining of livers from mice in the AAV8.Cre EtOH/CCl4 group and AAV8-null EtOH/CCl4 group (*p* = 0.26) even though it was significantly greater than AAV8.Cre pair-fed (0.10 ± 0.05, *p* = 0.025) and AAV8.null pair-fed (0.03 ± 0.01, *p* = 0.028) groups (Figure 4F). These data suggest that XIAP deletion does not exacerbate apoptosis and fibrosis in the EtOH plus CCl4 mouse model.

### Zonated Differential Expression of IAPs and Inhibition of SMAC Might Play a Role in Lack of *in vivo* Effect of XIAP Deletion

**FIGURE 5:**
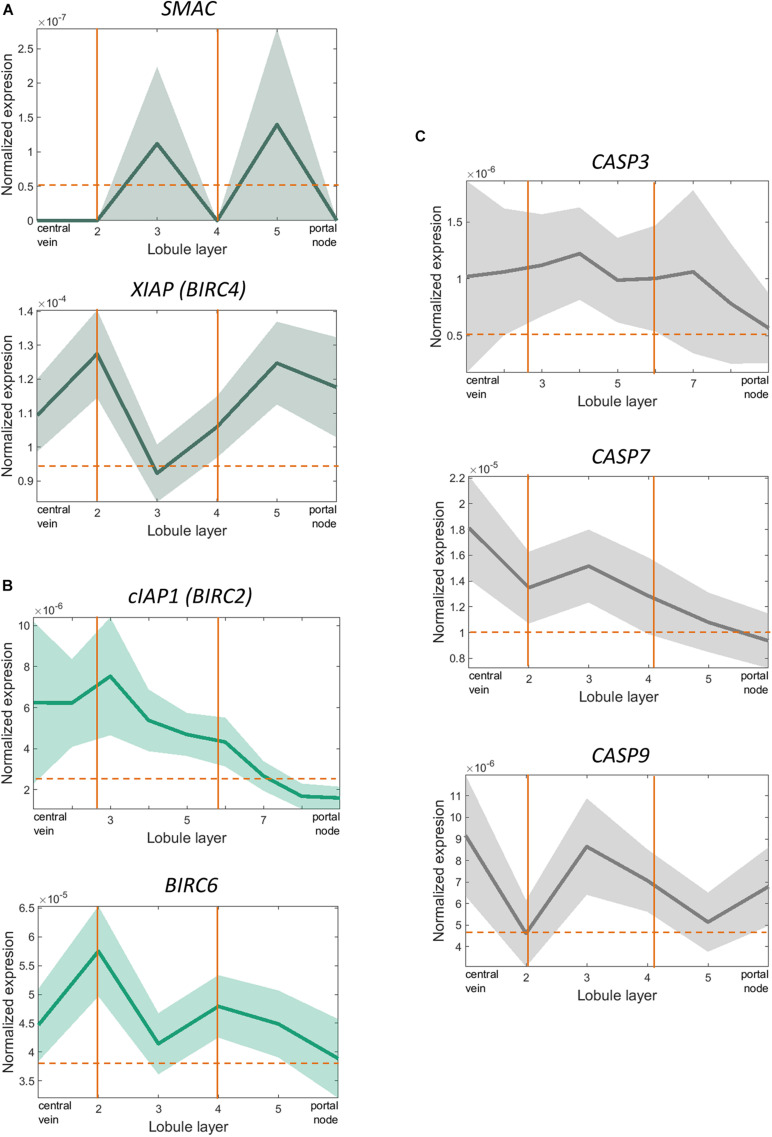
scRNA-sequencing showed significant overlapping zonation of *XIAP* and *SMAC* genes in human liver tissue. **(A)** *XIAP* and *SMAC* expression levels varied from peri-portal to peri-central hepatocytes and were significantly zonated peri-portally. Their expression levels peaked in the same layer of hepatocytes (*p* < 0.05). **(B)** cIAP1 (*BIRC2*) and Apollon (*BIRC6*) expression levels were zonated in the pericentral regions. **(C)** *CASP3*, *CASP7*, and *CASP9* expression levels varied from peri-portal to peri-central hepatocytes and were significantly zonated in the peri-central regions. The expression pattern did not overlap with *XIAP* or *SMAC*.

Even though *in vitro* evidence pointed towards an important role for XIAP in protecting against alcohol-induced cell death and inflammation, interestingly we did not see similar effects *in vivo*. As early-phase clinical trials are currently ongoing for XIAP inhibitors (NCT00882869, NCT00363974), we sought to understand the potential mechanism behind this lack of phenotype on a single cell level. We analyzed scRNA-seq performed on primary hepatocytes from human liver tissue to understand why hepatocyte-specific deletion of XIAP might have failed to exert a phenotypic effect in our *in vivo* experiments. As hepatocytes are known to perform different functions based on their spatial distribution, they were zonated along the portal-central axis of the liver lobule according to the established zonation landmarks (Massalha et al., 2020). SMAC, the only endogenous inhibitor of XIAP, as well as XIAP were distributed in a significantly zonated pattern in hepatocytes (Kruskal-Wallis Test with Benjamini-Hoeschberg correction-*p* < 0.05). XIAP (BIRC4) and SMAC expression were surprisingly identical to each other as we moved from peri-central to peri-portal layers (Figure 5A). On the other hand, other important IAP molecules such as cIAP1 (BIRC2) and Apollon (BIRC6) were significantly zonated in pericentral regions (Figure 5B). In line with these findings, molecules involved in the apoptotic pathway like caspase 3, caspase 7, and caspase 9, show a pericentrally zonated distribution pattern similar to cIAP1 and Apollon and opposite to that of XIAP and SMAC (Figure 5C). These spatial distribution patterns may highlight the role that compensation from IAPs expressing in different spatial areas as well as SMAC play in counteracting the effect of XIAP deletion.

## Discussion

XIAP has been widely recognized as a potent anti-apoptotic protein. Previous studies have shown that XIAP is a chemoresistance factor in mammalian cancer due to its anti-apoptotic function. XIAP targeting markedly enhances the cytotoxic activity of different cytostatic drugs in various tumor types (Cao et al., 2004; Hussain et al., 2017; Evans et al., 2018), and antagonists of XIAP have been tested in patients with different kinds of tumors in clinical trials (Dean et al., 2009; Carter et al., 2011; Mahadevan et al., 2013; Johnson et al., 2018; Ward et al., 2018). XIAP also plays a role in pathogen infection (Rigaud et al., 2011), inflammatory bowel disease (Worthey et al., 2011; Zeissig et al., 2015), and some hematological diseases (Marsh et al., 2010; Zhao et al., 2010; Speckmann et al., 2013). However, the function of XIAP in hepatocyte dysfunction associated with liver pathologies such as ALD is not known.

The present study provides insight into the biology of ALD showing that pharmacological and genetic deletion of XIAP in hepatocytes leads to divergent effects in *in vitro* and *in vivo* models and describes zonation patterns of IAPs as a potential mediator of this divergence. We utilized *in vitro* techniques using a widely used hepatoblastoma-derived tumoral hepatocyte cell line, HepG2, to understand the role of XIAP in alcohol-induced injury. We noted increased activation of caspases on deletion of XIAP. In the event of excessive caspase activity, such as in alcohol-induced injury, endogenous IAPs fail to control caspase activation which leads to excessive cellular apoptosis (Feldstein et al., 2003). Despite the known caspase-inhibitory function of XIAP, therapies harnessing the anti-caspase effects of XIAP are lacking due to poor understanding of its regulation and the ability to selectively modulate XIAP in hepatocytes. Furthermore, inflammation is a hallmark of alcohol-associated hepatitis (Liu et al., 2020; Sehrawat et al., 2020b). Increasing evidence points to extracellular vesicles (EVs) being paracrine signaling mediators that connect hepatocyte injury and macrophage activation in a variety of hepatic pathobiological processes (Verma et al., 2016; Liu et al., 2017). We show data supporting pro-inflammatory influence of these EVs from AH patients on M1-like macrophage activation. We and others have previously utilized EV release induction as a pathogenically relevant inflammatory marker (Arab et al., 2020b; Sehrawat et al., 2020a). We noted increased EV release on ethanol treatment from cells when XIAP was knocked down. This increase was prevented by XIAP overexpression *in vitro*. While these were interesting findings, HepG2 cell line is a hepatoblastoma-derived cell line. These cells are notoriously resistant to cell-death and have many inherent differences to primary hepatocytes (Wilkening et al., 2003; Gerets et al., 2012). These data thus led us to test these relevant findings in PMH exposed to alcohol-induced injury.

Interestingly, we found an unexpected divergent effect of XIAP deletion towards disease process in alcohol-induced injury in primary mouse hepatocytes. We observed a robust increase in apoptotic cell death in *Xiap* deleted mouse cells basally and an exacerbation on ethanol treatment. Caspase activation has been shown by us and others previously to contribute towards alcohol-associated liver disease development (Verma et al., 2016; Liu et al., 2017). We observed a reduction in inflammation in *Xiap* deleted PMH as well as reduced induction of inflammatory cross-talk between PMH and BMDMs. This observation is in line with previous findings in other chronic inflammatory gastroenterological disorders, such as inflammatory bowel disease (Pedersen et al., 2014). In fact, a number of receptors involved in immune response have been shown to signal through IAP ligases, including TNF-receptor family (e.g., CD40) pathways (Pedersen et al., 2014). Furthermore, bacterial products have been shown to induce inflammasome activation via activation of IAPs, including XIAP (Andree et al., 2014). These pathways are known to be involved in pathogenesis of ALD (Sehrawat et al., 2020b). These findings are especially important due to the complex pathophysiology around development and progression of ALD, as a disease exhibiting widespread apoptosis of hepatocytes as well as an aberrant inflammatory milieu and susceptibility to infections. Thus, we aimed to utilize *in vivo* alcohol-induced injury models to better understand these effects.

It has been reported that XIAP can promote accumulation of extracellular matrix and myofibroblasts leading to fibrosis in the lung (Ashley et al., 2015). Thus, we aimed to investigate whether *Xiap* deletion could exert anti-fibrotic effects in ALD mice models. There are several ALD mice models, including the chronic ethanol feeding model and the chronic binge model (Bertola et al., 2013; Mathews et al., 2014). However, both primarily induce steatosis and reactive oxygen species injury without severe hepatocyte apoptosis (Brandon-Warner et al., 2012; Mathews et al., 2014). To further enhance hepatocyte apoptosis, we modified the models by adding FAS agonist Jo2 and CCl4 model in XIAP hepatocyte specific deletion mice. The EtOH plus Jo2 model resulted in increased ALT, TUNEL staining, and cleaved-caspase 3 levels. However, hepatocyte-specific XIAP deletion did not show a substantial effect on liver injury, steatosis or inflammation in this model. These results are in accordance with a previous study which showed that the loss of XIAP alone did not increase FAS-mediated apoptosis in the liver (Jost et al., 2009). In EtOH plus CCl4 model, there was no difference in the TUNEL staining between the EtOH plus CCL4 group and the pair-fed plus olive oil group, which could be because CCl4 predominantly causes necrotic liver injury (Deshpande et al., 2016). Sirius Red staining and Western blot of collagen I demonstrated that EtOH plus CCl4 model induced increased fibrosis in mice livers. Surprisingly, hepatocyte-specific XIAP deletion did not show substantial effect on apoptosis and fibrosis in this model either. These unexpected observations however, are consistent with findings previously reported by other groups (Ashley et al., 2015; Edison et al., 2017). As an example, *Xiap* transgenic deletion does not attenuate bleomycin-induced fibrosis in mice, and mesenchymal cells isolated from these mice did not show increased Fas-mediated apoptosis (Ashley et al., 2015). Similarly, *cIAP1* and *cIAP2* knockout mice did not show any difference in the phenotype compared to wild-type mice (Conze et al., 2005; Conte et al., 2006; LaCasse et al., 2008). The absence of alterations in the phenotype of *Xiap*-deficient mice could be explained by the potential of other IAPs such as cIAP1 and cIAP2 to functionally compensate the genetic lack of *XIAP* expression. Indeed, it was demonstrated that *Xiap*-deficient mouse mesenchymal cells showed enhanced expression of cIAP2 (Ashley et al., 2015). Thus, even though all these IAPs, and especially XIAP can suppress cell death, their expression is differentially regulated in response to injury.

Hepatocytes in the liver are spatially distributed in highly organized structures, called liver lobules, and perform distinct functions (Massalha et al., 2020). In fact, different insults to the liver propagate injury in distinct patterns as well. Alcohol-induced liver damage is shown to initiate from pericentral areas and progress towards the periportal regions (Altamirano et al., 2014). Advent of novel technologies such as scRNA-seq has provided us with tools for the first time to dissect this variability in gene expression with such granularity. We utilized scRNA-seq from primary hepatocytes from human liver tissue in order to provide a potential explanation for these discrepancies observed between *in vivo* in IAP knockout disease models. We observed a significant periportal gene expression enrichment for XIAP in hepatocytes. The same pattern was observed for its only endogenous inhibitor, SMAC. On the other hand, other important IAPs such as *BIRC2* (cIAP1) and *BIRC6* (Apollon) were significantly zonated in the pericentral regions of the liver lobule. These regions had lower *SMAC* expression and it may be speculated that no inhibition of these IAPs via *SMAC*. Interestingly, caspases 3, 7, and 9 expression levels were also seen in pericentral and midlobular regions to support this hypothesis. Based on this, the failure of phenotype development in our *in vivo* models may partly be attributable to SMAC counteraction of XIAP in the intact liver. This is in contrast to isolated hepatocytes, where the deletion of XIAP, predictably, sensitized hepatocytes to the pro-apoptotic effects of ethanol. XIAP is also known to have ubiquitin E3 ligase activity, leading to proinflammatory signaling via receptor interacting protein 2 (RIP2) (Vucic, 2018), though, we observed similar activation of inflammatory pathways following XIAP deletion *in vivo*. Furthermore, the cytosolic form of SMAC, expressed consistently across human tissues and cancer cell lines, has been shown to be associated with tumorigenicity (Grimshaw et al., 2008; Paul et al., 2018). Receptor-interacting protein kinase 1 (RIPK1) polyubiquitination by IAPs has been shown to promote cytoprotection and cell survival in cancer cells via NF-κB activation (Pedersen et al., 2014). Supporting this, SMAC mimetics combined with caspase-8 inhibitors have been shown to be effective in some cancer cell lines (McCabe et al., 2014; Brumatti et al., 2016). Due to the complex roles of XIAP in regulating cell death and inflammation and the opportunity to augment SMAC function to promote cancer cell death, there are ongoing trials assessing the safety and efficacy profile of SMAC modulation in cancer patients. Thus, there might be a potential therapeutic relevance of targeting SMAC and XIAP together, in a chronic inflammatory and pro-apoptotic disease such as ALD which may also progress to hepatocellular carcinoma, to achieve desired effects. These are potential research questions that remain to be answered.

In conclusion, our findings suggest a divergent *in vitro* and *in vivo* phenotype for *Xiap* knockout mice in ALD models. There might be a compensation from other IAPs and a zonally dependent role for SMAC that prevented the development of an *in vivo* phenotype. Thus, targeting SMAC may also be important in addition to current efforts of targeting XIAP in treatment of ALD. Furthermore, with advancements in precision medicine, future analysis of IAP modulation should consider deciphering variable IAP expression profiles among patient populations to potentially translate these into personalized therapeutic agents.

## Data Availability Statement

The datasets presented in this study can be found in online repositories. The names of the repository/repositories and accession number(s) can be found below: GEO, Accession No. SE146409.

## Ethics Statement

The studies involving human participants were reviewed and approved by Mayo Clinic Institutional Review Board. The patients/participants provided their written informed consent to participate in this study. The animal study was reviewed and approved by Mayo Clinic IACUC (Approved protocol # A00005146-20; Approved protocol name: Molecular Mechanisms of Liver Fibrosis; PI: VS).

## Author Contributions

TS and VV contributed to study design, data acquisition, analysis and interpretation, and statistical analysis. LH, AN-C, GS, AM, SC, XL, TK, JC, and JA contributed to data acquisition. AN-C and SS contributed to data interpretation and writing of the manuscript. TS, AN-C, HM, and VS contributed to editing and finalizing the manuscript. HK contributed with XIAP f/f mice utilized for the studies. GG provided intellectual insight and contributed to editing and finalizing the manuscript. HM and VS contributed to study concept, design, funding and supervision, critical revision of the manuscript, and important intellectual content throughout the project. All authors read and approved the manuscript before submission.

## Conflict of Interest

The authors declare that the research was conducted in the absence of any commercial or financial relationships that could be construed as a potential conflict of interest.

## Footnotes

1 https://www.ncbi.nlm.nih.gov/geo

2 http://www.ncbi.nlm.nih.gov/geo/query/acc.cgi?acc=GSE146409

## Abbreviations

AAV8: adenovirus associated vector type 8
AH: alcoholic hepatitis
ALD: alcohol-associated liver disease
ALT: alanine aminotransferase
CCl4: carbon tetrachloride
CXCL1: C-X -C- motif chemokine ligand-1
EtOH: ethanol
EV: extracellular vesicle
HCC: hepatocellular carcinoma
IAP: inhibitor of apoptosis protein
IL1β: interleukin 1-beta
ORO: oil-red O
scRNA-seq: single cell RNA sequencing
SMAC: second mitochondria-derived activator of caspases
TNF-α: tumor necrosis factor-alpha
TUNEL: terminal deoxynucleotidyl transferase dUTP nick end labeling
XIAP: X-linked inhibitor of apoptosis protein.
